# Simplified Tai Chi Resistance Training versus Traditional Tai Chi in Slowing Bone Loss in Postmenopausal Women

**DOI:** 10.1155/2015/379451

**Published:** 2015-06-07

**Authors:** Huiru Wang, Bo Yu, Wenhua Chen, Yingzhi Lu, Dinghai Yu

**Affiliations:** ^1^Department of Physical Education, Shanghai Jiaotong University, 1954 Huashan Road, Shanghai 200030, China; ^2^School of Chinese Martial Arts, Shanghai University of Sports, 399 Changhai Road, Shanghai 200438, China; ^3^Department of Rehabilitation, Shanghai First People's Hospital, Shanghai Jiaotong University, 100 Haining Road, Shanghai 200080, China; ^4^School of Kinesiology, Shanghai University of Sports, 399 Changhai Road, Shanghai 200438, China

## Abstract

*Background.* This study examined whether simplified Tai Chi resistance training is superior to traditional Tai Chi in slowing bone loss in postmenopausal women.* Methods.* This prospective trial included 119 postmenopausal women (age: 52–65 years). Subjects were randomly assigned to participate in a traditional Tai Chi program (TTC, *n* = 40), a simplified Tai Chi resistance training program (TCRT, *n* = 40), or a blank control group (routine activity, *n* = 39). The TTC involved traditional Yang Style Tai Chi. The primary outcome was the change of lumbar bone mass density (L2–L4) at 12 months over the baseline. Femoral neck and Ward's triangle were also measured using dual-energy X-ray absorptiometry.* Results.* The L2–L4 density was significantly lower at 12 months in comparison to the baseline in the blank control group. In both the TCRT and TTC groups, the L2–L4 density was comparable to the baseline. There was a trend for less bone loss in the TCRT than in the TTC group. Similar findings were observed with femoral neck and Ward's triangle.* Conclusion.* Simplified Tai Chi resistance training could slow bone loss in menopausal women. The results also suggested, but did not confirm, superiority to traditional Tai Chi.

## 1. Background

Osteoporosis is an increasingly important public health problem as the world's population ages. Data from 2013 indicate that, in China alone, more than 69 million people have osteoporosis, including more than 15% of the population older than 50 years; another 210 million people have low bone mass that places them at risk of the disease [1]. In healthy individuals, bone loss starts at the age of 40 and accelerates after menopause in women.

Exercise is widely used to improve bone strength and counteract osteoporosis in menopausal women [2–5]. Several previous studies have shown that the ancient Chinese martial art Tai Chi is effective in this regard [6–8]. Programs derived from Tai Chi, such as Tai Chi Ball and Tai Chi Kung Fu Fan, can also improve bone mineral density and bone metabolic markers in middle-aged and elderly people, especially postmenopausal women [9, 10].

One challenge to wider adoption of Tai Chi among populations is that traditional sequences of movements are complex and lengthy [11]. Learning curve is slow; at-home practice is difficult. Efforts are needed to simplify Tai Chi exercises [12, 13].

We developed a simplified Tai Chi exercise program consisting of 4 basic modules, each consisting of 8 movements. Resistance was incorporated into the last module. The current study is a prospective trial that examined the potential benefit of this simplified Tai Chi program. The intervention lasted for 12 months (four 1 hr sessions per week). A positive control group practicing traditional Tai Chi program and a blank control group (routine activity) were included for comparison.

## 2. Materials and Methods

### 2.1. Subjects

Study subjects were recruited from healthy postmenopausal women in Shanghai, China, through advertisements placed in local newspapers and in community health centers. The study was approved by the Scientific Research Ethics Committee of Shanghai University of Sports (201206). All subjects signed informed consent forms and were told the purpose of the study and what they are expected to do during the study period.

Similar to a previous study [14], eligibility criteria included (1) menopause prior to at least 6 months and (2) age between 52 and 65 years. No subjects were employed in jobs with substantial physical demands.

Exclusion criteria included (1) a diagnose of any disease that affects bone metabolism; (2) a diagnosis of osteoporosis; (3) previous episode of suspected osteoporosis-related fracture; (4) a diagnosis of rheumatic diseases; (5) major diseases that affect the ability to exercise (e.g., severe hypertension, angina, or stroke); (6) subjects routinely taking calcium and/or vitamin supplements; (7) subjects who had been practicing Tai Chi for at least three months prior to the study.

### 2.2. Exercise Interventions

Enrolled subjects were randomized into three groups. Subjects in the blank control group were asked to maintain their routine living habits, not to participate in any health education related to osteoporosis, not to participate in any other exercise programs, and not to regularly consume any medication that could affect bone metabolism (e.g., calcium or vitamin A supplement).

Subjects in the traditional Tai Chi program (TTC) practiced traditional Yang style Tai Chi four times a week for 60 min each time, for a total duration of 12 months. Two out of the four sessions were group exercise under the supervision of coach Kaiyuan Wang, and the 2 remaining sessions were group exercise using a training video. Participation was recorded by the coach or group leaders. Each exercise session comprised 10 min of warming-up; 5 repetitions of a 6 min set of “42 type competition” actions (with 2 min rest in between), and 10 min of relaxing activities at the end.

Participants in the Tai Chi resistance training (TCRT) practiced simplified Tai Chi resistance training four times a week for 60 min each time. Two out of the four sessions were group exercise under the supervision of coach Anzhou Zhu, and the 2 remaining sessions were group exercise using a training video. Participation was recorded by the coach or group leaders. Each exercise session comprised 10 min of warming-up; 6 repetitions of a 5 min exercise (2 min rest in between); and 10 min of relaxing activities at the end.

To ensure subject compliance, diet and exercise were inquired verbally by the coach (in the 2 Tai Chi groups) or personnel of community health center (in the blank control group).

### 2.3. Bone Mineral Density Measurement

Bone mineral density in the lumbar L2–L4 region, femoral neck, and Ward's triangle was measured in all subjects at the baseline and at the end of the 12-month study. The measurement was carried out at Shanghai First People's Hospital using dual-energy X-ray absorptiometry.

### 2.4. Statistical Analysis

All analyses were conducted using SPSS 20.0. Data were examined with Shapiro-Wilk test to verify normal distribution. The primary outcome of bone mineral density change was compared to the baseline using paired Student's *t*-test. Absolute values in the 3 groups were analyzed using ANOVA.

## 3. Results

**Figure 1:**
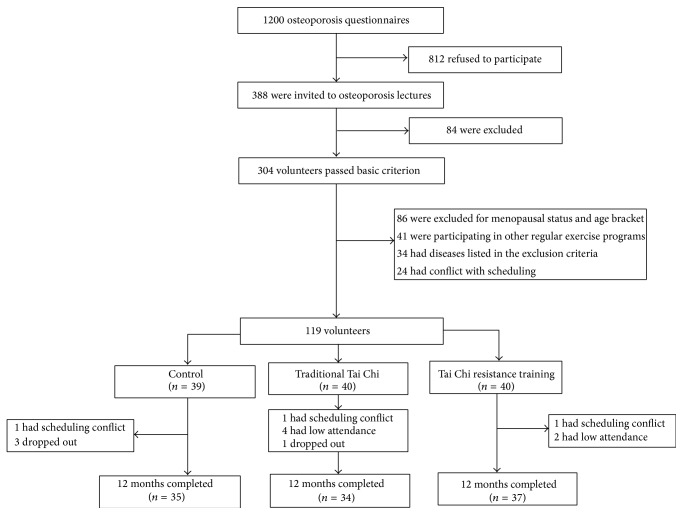
Screening, randomization, and completion of 12 months.

A total of 1,200 subjects were approached; 812 refused to participate. The remaining 388 subjects attended the initial training video session; 304 were willing to proceed with the study and were screened for eligibility. Among the 304 subjects, 86 were excluded for menopausal status and age bracket; 41 were participating in other regular exercise programs; 34 had diseases listed in the exclusion criteria; 24 had conflict with scheduling. A total of 119 subjects (age: 58.5 ± 3.5 years) were randomized, *n* = 39 for the blank control group and *n* = 40 for both the TTC and TCRT groups (Figure 1).

The three groups did not differ in height, weight, age, or bone mineral density at the baseline. The number of subjects who did not complete the 12-month study was 4, 6, and 3 in the blank control, TTC, and TCRT groups, respectively. No severe injury occurred during the study period (Table 1).

**Figure 2:**
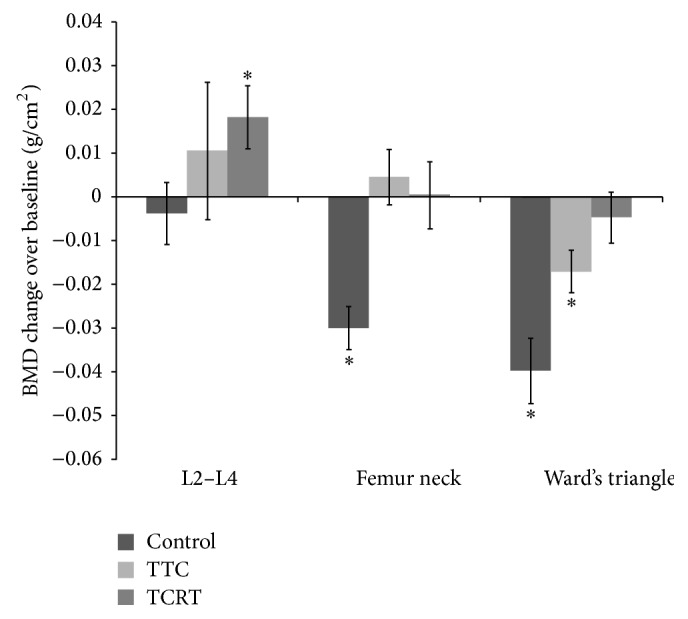
Change of bone mineral density in L2–L4, femur neck, and Ward's triangle over baseline.

Bone mineral density in all 3 anatomical regions followed normal distribution. Bone loss was significant in the blank control group, as reflected by a statistical reduction of BMD in femur neck at the completion of the 12-month study (0.81 ± 0.10 g/cm^2^ versus 0.84 ± 0.11 g/cm^2^ in the baseline; *p* = 0.00008), and similar finding was observed in Ward's triangle (0.63 ± 0.11 g/cm^2^ versus 0.67 ± 0.12 g/cm^2^; *p* = 0.00558). There was a significant upward trend of bone mineral density at L2–L4 area in TRCT group compared with baseline (1.10 ± 0.17 g/cm^2^ versus 1.08 ± 0.17 g/cm^2^; *p* = 0.01485). BMD of femur neck in both the TTC and TRCT groups as well as Ward's triangle in TRCT group did not differ significantly over the 12-month study period (Figure 2).

The absolute BMD did not differ significantly among the 3 groups, in any of the 3 anatomical regions. However, a statistically nonsignificant trend for higher L2–L4 BMD was noticed in the TRCT group (*p* = 0.06) (Table 2).

## 4. Discussion

Delaying bone loss of women after menopause is an important contribution to preventing osteoporosis-related risk (especially osteoporotic fractures). In recent years, several studies have confirmed that regular exercise has a role in prevention and treatment of osteoporosis. However some particular training methods such as resistance exercise sometimes cause adverse metabolic response [15–17]. Moderate-intensity endurance training normally includes jogging, stair climbing, and cycling. Generally, jogging and stair climbing are open kinetic chain exercises and they may increase the load on the joints to cause and aggravate osteoarthritis [18, 19]. Although cycling is closed kinetic chain exercise, it is mainly for muscular strength and endurance in the lower extremities and subject to field conditions and the specific equipment [20]. Tai Chi is characteristic of gentle, rhythmic movements and suitable for the elderly, and Tai Chi movement is considered closed kinetic chain justified with better safety.

Tai Chi has been shown in numerous studies to increase bone mineral density in postmenopausal women, but the complete traditional exercises are lengthy and complex, making them inaccessible to those who lack the time, commitment, or mobility to exercise in groups under the supervision of an instructor.

Traditional Tai Chi exercise programs can reverse this loss of bone mineral density. A case-control study involving 17 Chinese women in early postmenopause who practiced traditional Tai Chi for four years showed that mineral density in the lumbar region, proximal femur, and distal tibia was significantly higher than in a sedentary control group [21]. This suggests that traditional Tai Chi exercise can delay mass loss from weight-bearing bone in postmenopausal women. Similar results were reported in a randomized controlled trial involving 132 healthy Chinese postmenopausal women who practiced 108 styles of Tai Chi for 12 months [22]. That study found that bone loss between the distal tibial trabecula and cortical interval was 2.6- to 3.6-fold slower in the exercise group than in the sedentary control group. A cross-sectional study involving 99 Chinese women in early postmenopause found that practicing traditional complete Tai Chi exercise for more than 3 h per week for more than 3 years increased bone mineral density and improved neuromuscular function [23].

The ability of traditional Tai Chi to slow loss of bone mineral density in postmenopausal women is supported in several studies performed in the US and China [24–26], as well as in a recent meta-analysis including several randomized controlled trials [27].

Here we examine the potential of a simplified Tai Chi resistance training program to increase bone mineral density in such women, and we find the effects on density to be comparable to those with a complete traditional program. Of the five main branches of traditional Tai Chi [28], the Yang style is perhaps the most popular due to its gentleness and extensive stretching. Chen style, in contrast, requires more strength and involves more skipping movements; this style may consume the most energy of all five branches [29]. Our program of simplified Tai Chi resistance training program includes four Chen style actions for improving strength. It may be necessary to modify this exercise program, for example, by borrowing from gentler Tai Chi branches, in order to accommodate practitioners with significant mobility problems or frail health.

The Tai chi resistance training program is easier to learn and practice, and it can be performed at home within a limited space. Strength training and resistance exercises are generally effective at improving bone mineral density in postmenopausal women. Our simplified Tai Chi resistance training program includes the “pushing hands” movement, which is named Tai Chi Tuishou in China, which is similar to resistance exercises and so builds strength [30]. The simplified Tai Chi resistance training program is easy and enjoyable and it adds an element of social interaction, which makes it more interesting and beneficial for psychological and physical health. This exercise is also not limited by space and hardware facilities and it is readily accessible in community-based venues. So, this program is worthy of promotion to menopausal women to reduce bone loss.

## 5. Conclusions

Both traditional and simplified Tai Chi resistance training programs could decrease bone loss in postmenopausal women. Between the two programs, there seems to be an advantage for the simplified resistance program. The simplified Tai Chi resistance training program may be an effective alternative for practitioners with limited skill and mobility.

## Figures and Tables

**Table 1:** Baseline characteristics of TTC and TCRT.

Characteristic/bone region	Control (*n* = 39)	TTC (*n* = 40)	TCRT (*n* = 40)	*F *	*p *
Age, yr	58.54 ± 3.37	58.54 ± 3.37	57.93 ± 3.22	1.54	0.246
Height, cm	159.27 ± 4.84	159.27 ± 4.84	160.80 ± 4.37	1.09	0.236
Weight, kg	60.47 ± 8.31	60.47 ± 8.31	60.04 ± 6.65	1.51	0.126

Bone mineral density, g/cm^2^					
L2–L4	1.02 ± 0.14	1.03 ± 0.15	1.08 ± 0.17	1.67	0.0973
Femur neck	0.84 ± 0.11	0.84 ± 0.14	0.87 ± 0.12	1.13	0.2612
Ward's triangle	0.67 ± 0.12	0.66 ± 0.14	0.69 ± 0.13	1.12	0.2654

^*∗*^Values for characteristics showing a normal distribution are reported as mean ± SD, while values for other characteristics are reported as median (interquartile range).

**Table 2:** Bone mineral density after 12 months of intervention.

Characteristic/bone region	Control (*n* = 35)	TTC (*n* = 34)	TCRT (*n* = 37)	*F *	*p *
Bone mineral density after 12-month exercise intervention, g/cm^2^					
L2–L4	1.01 ± 0.13	1.04 ± 0.16	1.10 ± 0.17	1.88	0.0632
Femur neck	0.81 ± 0.10	0.83 ± 0.13	0.86 ± 0.12	1.07	0.2887
Ward's triangle	0.63 ± 0.11	0.64 ± 0.13	0.68 ± 0.14	1.54	0.1278

Difference from bone mineral density at baseline, g/cm^2^					
L2–L4	−0.0038 ± 0.0300	0.0105 ± 0.0361	0.0182 ± 0.0434	3.17	0.0464
Femur neck	−0.0300 ± 0.0388	0.0045 ± 0.0800	0.0004 ± 0.0281	4.54	0.0131
Ward's triangle	−0.0397 ± 0.0467	−0.0171 ± 0.0365	−0.0047 ± 0.0337	7.13	0.0013

^*∗*^Values for characteristics showing a normal distribution are reported as mean ± SD, while values for other characteristics are reported as median (interquartile range).
